# Accelerated sintering in phase-separating nanostructured alloys

**DOI:** 10.1038/ncomms7858

**Published:** 2015-04-22

**Authors:** Mansoo Park, Christopher A. Schuh

**Affiliations:** 1 Department of Materials Science and Engineering, Massachusetts Institute of Technology, 77 Massachusetts Avenue, Cambridge, Massachusetts 02139, USA

## Abstract

Sintering of powders is a common means of producing bulk materials when melt casting is impossible or does not achieve a desired microstructure, and has long been pursued for nanocrystalline materials in particular. Acceleration of sintering is desirable to lower processing temperatures and times, and thus to limit undesirable microstructure evolution. Here we show that markedly enhanced sintering is possible in some nanocrystalline alloys. In a nanostructured W–Cr alloy, sintering sets on at a very low temperature that is commensurate with phase separation to form a Cr-rich phase with a nanoscale arrangement that supports rapid diffusional transport. The method permits bulk full density specimens with nanoscale grains, produced during a sintering cycle involving no applied stress. We further show that such accelerated sintering can be evoked by design in other nanocrystalline alloys, opening the door to a variety of nanostructured bulk materials processed in arbitrary shapes from powder inputs.

## Abstract (web summary)

In sintering, powders of small grains are packed together to form shapes or grain structures that cannot be achieved by melt casting. Here, the authors demonstrate the fast sintering of a nanostructured alloy at low temperatures, preserving its nanoscale grain structure.

## Graphical abstract

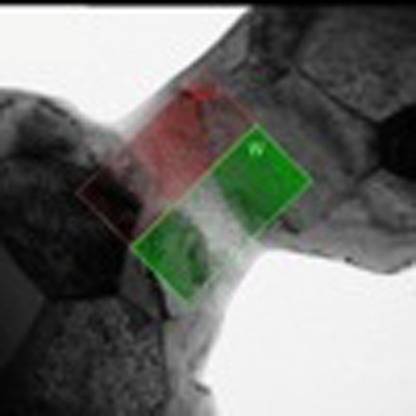

Although sintering is a common processing method for manufacturing bulk polycrystalline materials, it can often require long time-at-temperature cycles that pose problems for structural stability, for example, grain growth. In fact, although powder processing and sintering have long been studied as a route to achieve bulk nanocrystalline materials^1^,^2^,^3^,^4^,^5^,^6^,^7^,^8^, it is a challenge to use enough of a thermal cycle to remove all the porosity without also seeing large changes in grain size. So-called ‘accelerated' sintering techniques, such as activated sintering^9^,^10^,^11^ or liquid phase sintering^12^,^13^, have been used for decades to lower the sintering temperature and reduce the cycle time for sintering, but these methods do not apply to the synthesis of nanocrystalline materials. Here we show that accelerated sintering is possible in some nanocrystalline alloys that are designed to exhibit nanoscale phase separation, which in turn leads to a dual-phase structure that accelerates sintering.

## Results

### Nanocrystalline tungsten alloy powders

**Figure 1:**
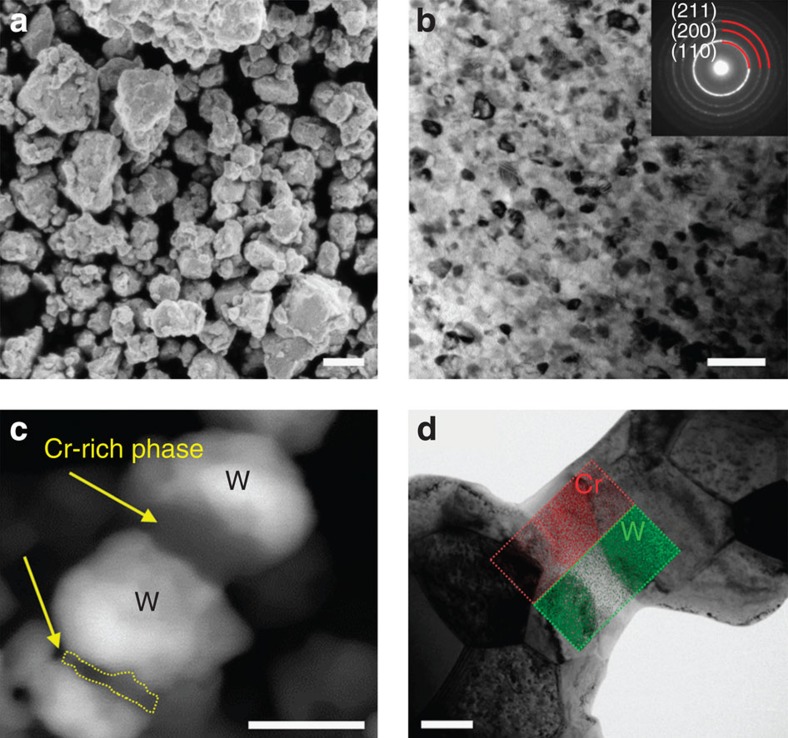
Pre- and postsintering microstructures of W-15 at% Cr alloy. (**a**) Scanning electron microscopy (SEM) image of as-milled tungsten alloy powder delineates micron-sized particles (scale bar, 1 μm). (**b**) The bright-field transmission electron microscopy (TEM) image shows the alloy after 20 h of high-energy milling, with nanoscale grains of about 13 nm characteristic size. The selected area diffraction pattern (inset) is indexed as being from a BCC solid solution (scale bar, 50 nm). (**c**) SEM in back-scatter mode reveals a chromium-rich phase forming necks between the compact particles on heating up to 1,200 °C (scale bar, 500 nm). (**d**) A direct visualization of a Cr-rich neck adjacent to W-rich particles is shown in the bright-field TEM image with W and Cr elemental maps (superimposed on the micrograph) using scanning TEM with energy dispersive spectroscopy (STEM-EDS) (scale bar, 200 nm). BCC, body-centred cubic.

We first mechanically alloyed W with 15 at% Cr using a high-energy ball mill. The resulting powder particles are micron size in diameter as shown in Fig. 1a (also see Supplementary Fig. 1), each particle being much larger than the average grain size of about 13 nm, as shown in the transmission electron microscopy (TEM) micrograph in Fig. 1b; each powder particle is polycrystalline with nanoscale grains^14^,^15^,^16^, which is an important distinction as compared with, for example, nanopowders, where every particle is of nanometre scale dimension and typically is a single crystal, and where some interesting sintering phenomena have also been observed^17^. The selected area diffraction pattern shown in the inset of Fig. 1b exhibits a Debye–Scherrer ring indexed as being from a body-centred cubic solid solution, which is in agreement with separate X-ray diffractometry (XRD) data (Supplementary Fig. 2). Although chromium has almost no equilibrium solubility in tungsten at room temperature^18^, high-energy ball milling is widely known to achieve supersaturation^19^,^20^,^21^ and the Cr is fully dissolved in W here; this supersaturated solution is thus poised to phase separate on heating.

**Figure 2:**
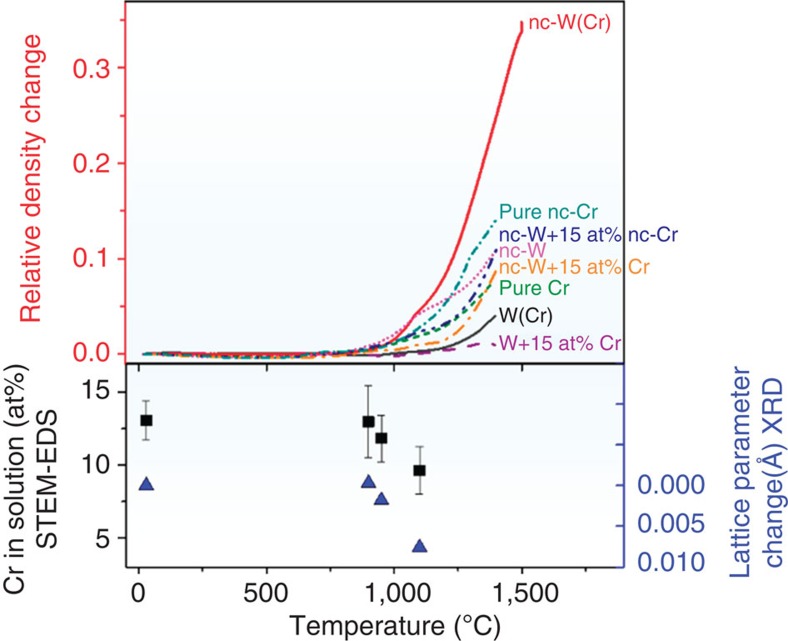
Changes in density and particle properties on heating. Relative density changes are from TMA measurements, chromium content dissolved in the powder particles is measured by STEM-EDS and the lattice parameter change of the BCC tungsten-rich phase is from X-ray diffraction (XRD), and each are shown as a function of temperature. The TMA data are also directly compared with the series of control experiments. Phase separation sets on at about 950 °C, which is also the point at which sintering accelerates. The error bars correspond to the s.d. of >10 different composition measurements on a single specimen. BCC, body-centred cubic.

From the as-milled powder, cylindrical compacts were formed by cold uniaxial pressing, and pressureless sintering was conducted while measuring the change in density as a function of time and temperature using a thermomechanical analyser (TMA) operated under flowing high-purity argon gas including 3% hydrogen. As shown in Fig. 2 for a typical experiment involving heating at a constant rate of 10 °C min^−1^, the compact began to noticeably densify at ∼950 °C, lower than the ∼1,100–1,200 °C at which liquid phase or conventional activated sintering generally sets on in tungsten, and even lower than the normal sintering onset for pure chromium of the same particle size, whether nanocrystalline (cyan line) or not (green line). By the time 1,500 °C is reached at this ramp rate (after a total time of 155 min.), the compact is nearly fully dense (>98%), although this cycle involved no external applied pressure.

### Conditions for rapid densification

The onset of sintering at 950 °C and the rapid rate of sintering seen thereafter in Fig. 2 are apparently triggered by the combination of two features of our powders: (i) nanocrystallinity within the microscale powder particles and (ii) alloy supersaturation that leads to phase separation on heating. This is established by the series of control experiments shown by the lines in Fig. 2. All of the powders used in these control experiments are prepared with similar micron size powder particles to the W–Cr powder described above, and thus are comparable in terms of the driving force for sintering and the length scales for mass transport required to achieve densification. More details are available in the Methods section. These control experiments illustrate a lack of significant densification in W powders that are milled to a nanocrystalline state without a Cr addition (magenta), and in powders that contain the required Cr content but which are not nanocrystalline supersaturated solid solutions (dark grey, purple, orange and dark blue). The dark grey line is significant because it shows data for supersaturated W-15 at% Cr powders prepared through a quenching route, and verifies that a supersaturated solid solution alone is insufficient to trigger densification if the powders are not also nanostructured.

The above data thus show that accelerated sintering occurs in this system, but only when it is prepared as a supersaturated solid solution with a nanoscale polycrystalline structure inside each powder particle. This effect may be traced to unique structural changes that occur in such a powder during the sintering cycle. As shown in the scanning electron microscopy (SEM) micrographs in Fig. 1c, a chromium-rich phase precipitated from the supersaturated nanocrystalline tungsten on heating, forming necks between the compact particles. A direct visualization of a Cr-rich neck adjacent to W-rich particles is shown in Fig. 1d, where scanning TEM with energy dispersive spectroscopy (STEM-EDS) measurements of local composition are superimposed on the micrograph. These images all illustrate that the metastable solid solution of W(Cr) decomposes on heating, and the Cr-rich phase precipitates within the particles (see Supplementary Note 1 and Supplementary Figs 3 and 4), but also, importantly, at the particle surfaces and interparticle necks. That such surface and neck sites are thermodynamically favourable for second phase nucleation and growth is documented in other systems as well, such as Cu-In and Ag-Au^22^,^23^. Owing to the nanocrystalline grain size within the powders, there are ample short-circuit diffusion pathways that allow the Cr out of the particle centres to decorate their surfaces.

### Kinetics of nanophase separation sintering

The above observations suggest a possible explanation for the rapid sintering in this W–Cr alloy: if a second phase precipitates, decorates particle surfaces and interparticle necks, and thusly provides new and more rapid diffusional transport pathways, then sintering may be expected to accelerate. The correlation between sintering and phase separation is made more explicit using STEM-EDS and XRD on compacts quenched partway through the densification cycle of Fig. 2, as shown by the solid black and blue data points in that figure. The STEM-EDS results present an explicit measurement of the Cr content in the powder particles, which is found to begin decreasing as the phase separation occurs. The XRD data are analysed to present the body-centred cubic lattice parameter for the W-rich phase, and this too begins to change on heating as the phase separation occurs and Cr is lost from the W-rich particles. What is noteworthy in these two data sets is that they both illustrate that phase separation sets on at about 950 °C, which is the same point at which sintering accelerates.

**Figure 3:**
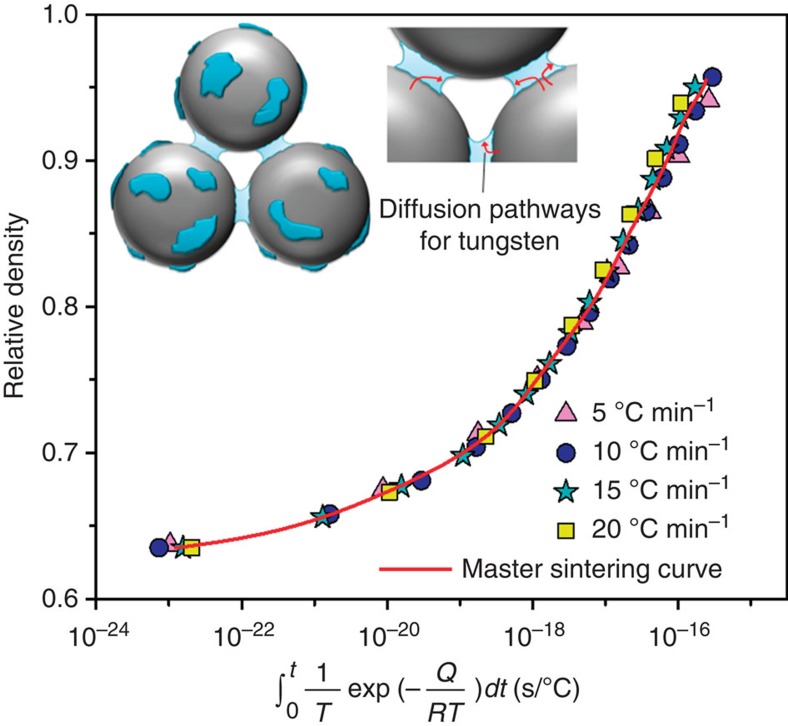
Master sintering curve and heating profiles of W-15 at% Cr with a schematic structure for nanophase separation sintering in the inset. All of the heating profiles, using several constant heating rates of 5, 10, 15 and 20 °C min^−1^, collapse onto a single curve at a sintering activation energy of 373 kJ mol^−1^, which reasonably matches the activation energy for diffusion of W in Cr, but is not consistent with any other bulk diffusional process in the W–Cr system.

We expect that the Cr-rich phase that precipitates and forms interparticle necks should be a rapid diffusional transport layer in this system: the melting point of Cr is much lower than that of W and diffusion in the Cr-rich phase is therefore faster^24^. What is more, Cr has a high solubility for W (∼15 at% at 1,200 °C (ref. 18), in line with STEM-EDS observations of local composition in Fig. 1d), and the diffusion of W through Cr is quite rapid at these temperatures^24^. Thus, once it is ejected from the supersaturated solution, the Cr-rich phase should be capable of dissolving and transporting W as well. Densification thus may occur by the transport of W (as well as Cr) from within particles into the Cr-rich neck region and outward to the neck edges to accommodate filling of the open space between the particles (Fig. 3 inset).

To evaluate possible rate-limiting kinetic processes that control densification in this system, we assessed the sintering activation energy using the master sintering curve method^25^ (see Supplementary Note 2). This is a method of normalizing sintering profile curves such as that shown in Fig. 2, but acquired over a range of heating rates. Figure 3 shows a series of such heating profiles at several heating rates (raw data provided in the Supplementary Fig. 5), with a normalized *x* axis of 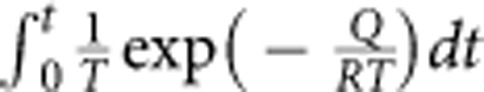, where *Q* is sintering activation energy, *R* is the gas constant, *T* is temperature and *t* is time. As shown in Fig. 3, all of our experimental data for the W-15 at% Cr system collapse onto a single curve given a best-fit sintering activation energy of 373 kJ mol^−1^. While this apparent activation energy is probably reflective of many processes occurring at once over the range of temperatures investigated, it is interesting that the value is very close to the activation energy for diffusion of tungsten in chromium, 386±33 kJ mol^−1^ (ref. 24), and is very different from both that for self-diffusion of W (550–670 kJ mol^−1^)^26^ that normally controls sintering of W, from that for self-diffusion of Cr (442 kJ mol^−1^)^27^, and even farther from that for Cr diffusion in W (547 kJ mol^−1^)^28^, indicating that the flow of Cr itself is not the source of the enhanced densification. Our examination of the morphology of the Cr phase at interparticle necks (Supplementary Note 3) also shows that this phase remains roughly the same scale throughout densification, also suggesting that the mechanical flow of Cr is not the source of the enhanced densification. The kinetics are thus consistent with W diffusion through Cr as being a kinetically rate-limiting process for sintering, and are not consistent with any other bulk diffusional process being dominant. The rates of sintering that might be expected if densification were dominated by W diffusion through Cr are also reasonably in line with those measured here (see Supplementary Note 4 and Supplementary Fig. 7). And of course, with the scale of the internal structure being of nanometre dimensions, short-circuit diffusion on interfaces and surfaces almost certainly also contributes to the structural evolution during sintering of such samples.

## Discussion

Although accelerated sintering schemes usually involve the introduction of rapid transport paths, our observations for the nanocrystalline W–Cr system present clear distinctions from other such sintering methods, including seed-assisted sintering, liquid phase sintering and solid-state activated sintering. First, so-called ‘seed-assisted sintering' involves a second phase that nucleates during sintering, but makes use of the nucleated phases in a structural way; a high number density of nucleated seeds prohibit the formation of a micrometre scale phase, which would significantly discourage sintering^29^,^30^. By contrast, nanophase separation sintering as we report here involves a nucleated phase that accelerates densification kinetics. Second, the chromium phase we observe in Fig. 1d is crystalline and not molten at any temperature studied here; at our composition, the liquid phase becomes viable only around 2,800 °C, and even pure Cr does not melt until 1,863 °C^18^. This system thus cannot benefit from accelerated sintering by liquid phase formation as in liquid phase sintering. Our measurements on the thickness of the Cr-rich interparticle necks (Supplementary Fig. 6) also verify that the mechanical deformation of the Cr phase is not the cause of the accelerated densification. Third, the Cr phase domains here are thick, and unlike the disordered grain boundary film (∼1 nm)^9^ such as forms and provides the rapid transport pathway in conventional solid-state activated sintering. In fact, a nanometre thick grain boundary film cannot be stabilized at such a low sintering temperature in W–Cr alloys, since the free energy penalty for its formation would not be compensated by a reduction in interfacial energy^9^,^10^.

**Figure 4:**
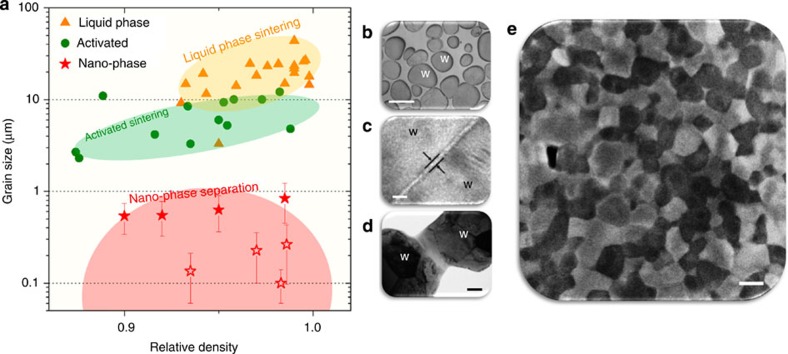
Comparison of nanophase separation sintering with liquid phase sintering and activated sintering of tungsten alloys. (**a**) Grain size as a function of relative density achieved using each sintering method shows that nanophase separation sintering lends itself to the production of ultrafine-grained material. Further comparison is illustrated with typical microstructures of (**b**) liquid phase sintering^13^, in which W particles are embedded in a liquid matrix that is the rapid transport path (scale bar, 80 μm) (**c**) activated sintering^10^, in which the grain boundary has a film on it that is a rapid transport path, and (scale bar, 2 nm) (**d**) nanophase separation sintering, in which the separation of the supersaturated solution decorates the interparticle necks with a second solid phase that is a rapid diffusion pathway (scale bar, 200 nm). The error bars correspond to the s.d. of more than 1000 different grains on a single specimen. (**e**) SEM image of a bulk (6 × 4 mm right cylinder) nanocrystalline W–Ti–Cr alloy shows a grain size of about 100 nm at nearly full density (scale bar, 100 nm).

Further comparison of nanophase separation sintering with liquid phase sintering and solid-state activated sintering is facilitated by Fig. 4, which compares studies that all employ powder particles with sizes in the range of 0.2–11 μm (Supplementary Table 2; Supplementary Fig. 11). Not only is nanophase separation sintering suitable specifically for nanostructured alloys, the addition of second phases and alloying elements is generally useful to retain nanocrystalline structures during a thermal cycle^31^,^32^,^33^. This methodology thus lends itself naturally to the production of fine-grained material, and in Fig. 4 our data for W alloy sintering attain much smaller grain sizes at comparable densities as compared with the other methods (although not all of the prior studies necessarily aimed to achieve fine grains).

The data sets marked by solid red stars in Fig. 4 all use a constant heating rate to a relatively arbitrary maximum temperature in a single alloy (W-15 at% Cr); further optimization of alloy composition as well as temperature–time cycle should permit a large measure of control over grain sizes in the ultrafine-to-nanoscale range in full density sintered products. For example, the red empty stars show related, more optimized alloys of W–Ti–Cr, also sintered without applied pressure. Here Ti is added because it promotes stabilization of the grain structure^31^. The final sintered structure of W-35Ti-10Cr (at%) is shown in Fig. 4e, reflecting nearly full density and a grain size of 100 nm. This particular sample had bulk dimensions of 6 mm diameter and 4 mm height; we are not aware of any prior nanocrystalline alloy with such a combination of full density and fine grains produced in bulk through pressureless sintering of powders.

In principle, the enhanced sintering revealed above in the W–Cr system may be widely accessible in other alloy systems; the basic requirements are a system that (i) can be prepared as supersaturated microscale powder with a nanoscale grain size (for example, by high-energy milling), (ii) will phase separate at a sintering temperature of interest, producing (iii) a fast transport layer on particle surfaces and necks. As a second example, we accelerated the consolidation of chromium with an addition of nickel through nanophase separation sintering. The system exhibited accelerated densification, and micrographs with local chemical mapping show that nickel precipitates around chromium necks (Supplementary Fig. 9). The sintering activation energy of Cr-15 at% Ni was measured as 258 kJ mol^−1^, close to the activation energy for diffusion of chromium in nickel, 272 kJ mol^−1^ (ref. 34), which is consistent with the precipitated Ni being a transport path for Cr. A detailed analysis of the Cr–Ni system is available in the Methods section.

Powder consolidation has long been viewed as a promising route to form bulk nanocrystalline and ultrafine-grained materials, but the challenges associated with rampant grain growth^35^ and significant residual porosity^36^ have delayed progress. To overcome such limitations, the field has seen a focusing tendency towards rapid consolidation methods assisted by large applied pressures^36^,^37^,^38^ or pulsed electric current^39^,^40^, although limitations on component size and shape, as well as cost considerations, present complications for the broad usage of these techniques. It is our hope that nanophase separation sintering, as a general new approach to accelerate sintering even in the absence of external forces, may broaden the opportunity for powder-route fabrication of bulk ultrafine and nanocrystalline alloys.

## Methods

### Powder processing

Average particle size (APS) 1–5 μm W powder (99.9% purity), APS <10 μm Cr powder (99.2% purity), APS 2–3 μm Ni powders (99.9% purity), and −150 mesh Ti powder (99.9% purity) were used in this study. W and W alloy (W-15 at% Cr, W-35Ti-10Cr), and Cr alloy (Cr-15 at% Ni) powders were produced by mechanical alloying in a SPEX 8000 high-energy mill using tungsten carbide media and a ball-to-powder ratio of 5 to 1, with 1 wt% stearic acid as a process control agent. All synthesized powders used the same milling procedures except for milling time: 20 h for W-15 at% Cr, 30 h for W-35Ti-10Cr and 15 h for Cr-15 at% Ni; these times were arrived at through experimentation to achieve full dissolution of solutes, while minimizing impurity contamination. Also, in the spirit of controlling impurities to the extent possible, all of the processing steps in this work were conducted in highly purified atmospheres. For a typical powder of W-15 at% Cr, we used EDS over a broad area in an SEM to verify that the contamination by Co (from the carbide milling equipment) was <1 wt%, while the pickup of tungsten carbide explicitly evaluated by XRD (Supplementary Fig. 2a) was <3 wt%. To counter the possibility of native oxide formation, the sintering cycle was conducted in a reducing atmosphere, as also described below. The particle sizes of all powders including those used in control experiments were measured using a laser diffraction particle size analyser from Horiba.

Supplementary Fig. 2a shows XRD patterns of W-15 at% Cr at different milling times. The main diffraction peak for chromium located at 44.4° disappears after about 4 h milling as shown in Supplementary Fig. 2b, which indicates that chromium is fully dissolved into tungsten. Tungsten carbide, picked up from abrasion of the milling media, starts to appear after 4 h of milling; the amount of tungsten carbide after 20 h, as assessed by Rietveld refinement, is around 1–3 wt% . Powders were compacted at a pressure of 360 MPa into 6 mm diameter and 3–4 mm high cylindrical disks. Each green compact was heated at a constant rate in flowing Ar+3% H_2_ using a TMA from Netzsch Instruments to measure its *in situ* length changes. The heating rates employed were 5, 10, 15 and 20 °C min^−1^. The force on the pellet from the alumina push-rod of the TMA was 100 mN. The W–Ti–Cr samples were processed in the same general manner as above, and all samples including those used for control experiments were sintered without applied pressure under a constant heating rate to 1,350–1,500 °C, followed by rapid cooling under flowing gas after the target temperature was reached.

### Micro- and nanostructure characterization

XRD patterns were measured using a PANalytical X'Pert Pro diffractometer using Cu-Kα radiation at 45 kV and 40 mA. All alloyed powders were scanned from 30 to 120° using a step size of 0.0167° and time per step of 90 s. The phases present, lattice parameters and grain sizes were assessed by Rietveld refinement. An XL30 Environmental FEG SEM from Philips and FEI Helios were used for imaging of the powders. The samples for TEM were prepared by a Fischione ion mill maintained at −110 °C by liquid nitrogen. Bright-field images and diffraction patterns were acquired using a JEOL 2010F TEM, and EDS was used to construct elemental maps and perform local composition measurements.

### Control experiments in the W–Cr system

We designed the series of control experiments in Fig. 2 to establish that nanophase separation sintering only occurs when powders with both nanocrystalline internal grain sizes and alloy supersaturation are used. The control samples were intended to systematically test various W–Cr materials featuring nanocrystallinity or supersaturation, but not both. All controls have micron-sized particles (Supplementary Table 1) in order to remove particle size effects on the driving force and kinetics of sintering. Each alloyed powder was compacted and sintered following the same procedures described above.

First, pure nanocrystalline W (labeled nc-W in Fig. 2) was mechanically milled in the SPEX 8000 high-energy mill for 20 h using tungsten carbide media and a ball-to-powder ratio of 5 to 1, with 1 wt% stearic acid as a process control agent. The resulting sample had a grain size of 10 nm as revealed by Rietveld refinement, but no Cr, and thus met the condition of being nanocrystalline, but was not supersaturated. This powder was then compacted into 6 mm diameter and 3–4 mm high cylindrical disks of 0.62 relative density.

Second, nanocrystalline W with 15 at% Cr (not dissolved; labeled nc-W+15 at% Cr in Fig. 2) was produced by adding pure Cr powder to pure nanocrystalline W (produced by high-energy milling for 20 h) using a dry mixing method; 15 at% Cr was mixed with nanocrystalline W without milling or mechanical alloying, for ∼15 min. The resulting sample comprised W with a grain size of 10 nm as revealed by Rietveld refinement, and contained chromium, but not in an alloyed or supersaturated condition; this sample thus featured nanocrystallinity but not supersaturation. This powder was then compacted into 6 mm diameter and 3–4 mm high cylindrical disks of 0.63 relative density.

Third, W-15 at% Cr unalloyed and without nanostructure (labeled W+15 at% Cr in Fig. 2) was produced by dry mixing 15 at% Cr with W for ∼15 min without mechanical alloying or milling. The resulting sample was a mixture of W-15at% Cr, but had neither nanoscale grain structure nor supersaturation. This powder was then compacted into 6 mm diameter and 3–4 mm high cylindrical disks of 0.67 relative density.

Fourth, nanocrystalline W with 15 at% nanocrystalline Cr (not dissolved; labeled nc-W+15 at% nc-Cr in Fig. 2) was produced by adding nanocrystalline Cr powder to pure nanocrystalline W powder, both produced by high-energy ball milling for 20 h and then dry mixing. The resulting sample comprised W particles with a grain size of 10 nm and Cr particles with a grain size of 17 nm as revealed by Rietveld refinement; it was nanocrystalline but not in an alloyed or supersaturated condition. This powder was then compacted into 6 mm diameter and 3–4 mm high cylindrical disks of 0.65 relative density.

Fifth, supersaturated W-15 at% Cr (labeled W(Cr) powder in Fig. 2) were produced by mechanical milling in a SPEX 8000 high-energy mill for 30 min using tungsten carbide media without any process control agent. The resultant powder was then sealed in a quartz tube, first evacuated to 10^−6^ Torr using a turbo pump and then backfilled with high-purity argon gas to 120 Torr. The sealed ampoule was annealed in a furnace at 1,400 °C for 20 h and then quenched. The resulting powder was a supersaturated W(Cr) solution, but with a coarse grain size in excess of 1 μm; it was supersaturated with chromium but not nanocrystalline. This tungsten solid solution powder was then compacted into 6 mm diameter and 2–3 mm high cylindrical disks of 0.65 relative density.

Sixth, pure Cr with a conventional microscale grain structure was produced by compacting powder into 6 mm diameter and 3–4 mm high cylindrical disks of 0.67 relative density.

Finally, nanocrystalline Cr (labeled nc-Cr in Fig. 2) was produced by mechanically milling pure chromium in the SPEX 8000 high-energy mill for 20 h using tungsten carbide media and a ball-to-powder ratio of 5 to 1, with 1 wt% stearic acid as a process control agent. The resulting sample had a grain size of 17 nm as revealed by Rietveld refinement; it was nanocrystalline but contained no alloying additions. It also contained no tungsten and provides a limiting case if the kinetics of densification were dominated by transport in the low melting point Cr phase in W–Cr systems. This powder was compacted into 6 mm diameter and 3–4 mm high cylindrical disks of 0.66 relative density.

### Sintering of larger specimens of W-15 at% Cr

A bulk specimen of W-15 at% Cr, of 15 mm diameter and 11 mm height, was sintered in a conventional high temperature furnace under Ar+3% H_2_ atmosphere. The sample shown in Supplementary Fig. 8 achieved full density under the same conditions specified in the paper, verifying that accelerated sintering is possible in this system at larger sample sizes and in a conventional furnace.

### Nanophase separation sintering in the Cr–Ni system

The Cr–Ni system was also studied as a second candidate to exhibit nanophase separation enhanced sintering. We accelerated the consolidation of chromium with additions of 5 and 15 at% Ni. The system exhibited accelerated densification as shown in Supplementary Fig. 9a, and micrographs show that nickel precipitates around chromium necks as shown in an SEM micrograph with the inset EDS map showing the local nickel content (Supplementary Fig. 9b). The master sintering curve method was employed to assess the sintering activation energy. The heating profiles employed for nanocrystalline Cr-15 at% Ni with 3, 5, 10, 15 and 20 °C min^−1^ heating rates are shown in Supplementary Fig. 10a. All of our experimental data for the Cr-15 at% Ni system collapse onto a single curve given a best-fit sintering activation energy of 258 kJ mol^−1^ as shown in Supplementary Fig. 10b, aligning with the activation energy for diffusion of chromium in nickel, 272 kJ mol^−1^ (ref. 34) and very different from that for self-diffusion of Cr (442 kJ mol^−1^)^27^ that normally controls sintering of Cr.

## Author contributions

M.P. and C.A.S. proposed the idea and designed the experiments. M.P. conducted all experiments and co-wrote the paper. C.A.S. provided guidance and co-wrote the paper. All authors analysed the data, discussed the results and reviewed the manuscript.

## Additional information

**How to cite this article:** Park, M. & Schuh C. A. Accelerated sintering in phase-separating nanostructured alloys. *Nat. Commun.* 6:6858 doi: 10.1038/ncomms7858 (2015).

## Supplementary Material

Supplementary Information Supplementary Figures 1-11, Supplementary Tables 1-2, Supplementary Notes 1-4 and Supplementary References
