# Supplementary material for: Accelerated sintering in phase-separating nanostructured alloys
Source: Nat Commun. 2015 Apr 22;6:6858. doi: 10.1038/ncomms7858 (PMC4423263; doi:10.1038/ncomms7858)
Supplement: Supplementary Information — Supplementary Figures 1-11, Supplementary Tables 1-2, Supplementary Notes 1-4 and Supplementary References [file ncomms7858-s1.pdf]

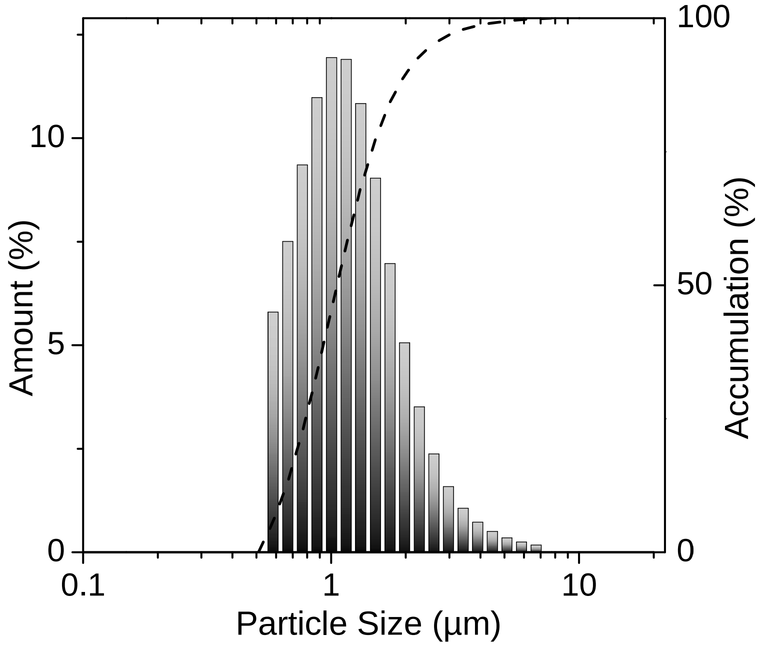

Supplementary Figure 1. The particle size distribution of W-15 at% Cr after 20 hours milling.

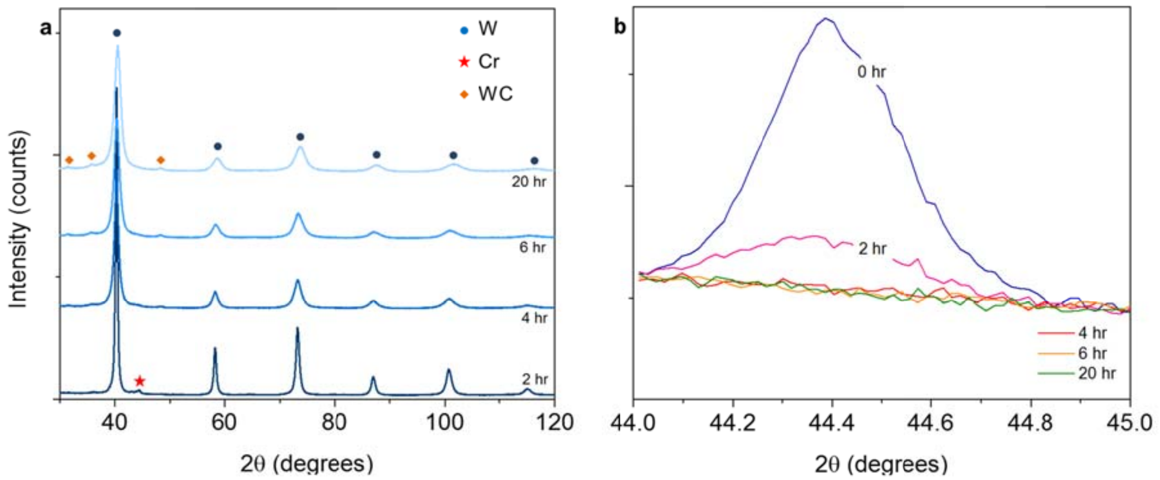

Supplementary Figure 2. a,b, X-ray diffraction patterns of W-15 at% Cr at different milling times **(a)** in the 2θ range between 30 and 130 °, and **(b)** 44 and 45 °.

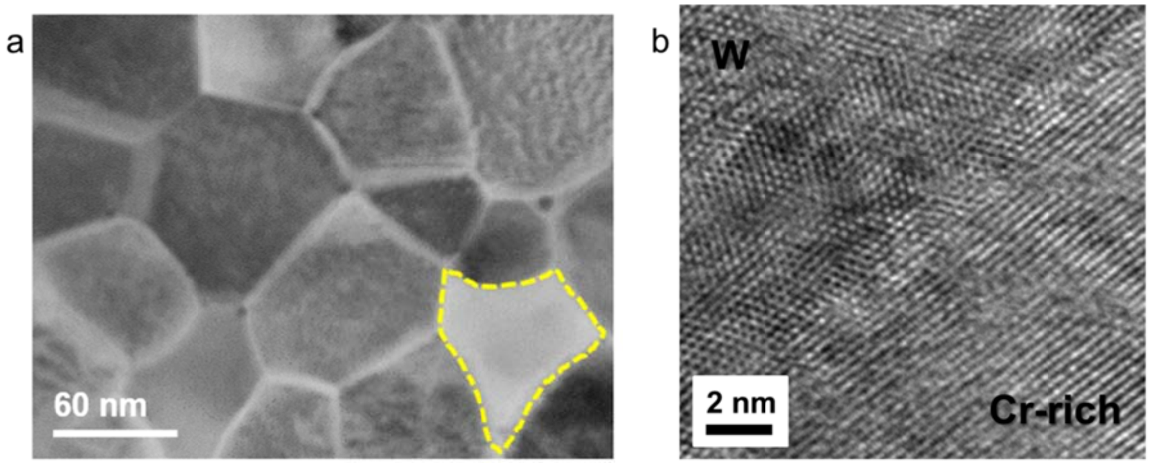

Supplementary Figure 3. a, A dark-field STEM image of the interior of a powder particle in a W-15 at% Cr alloy heated to 1400 °C, with one Cr-rich phase domain among W-rich grains being highlighted. **b,** a HR-TEM micrograph showing the crystalline interface between W-rich and Cr-rich domains at a particle neck.

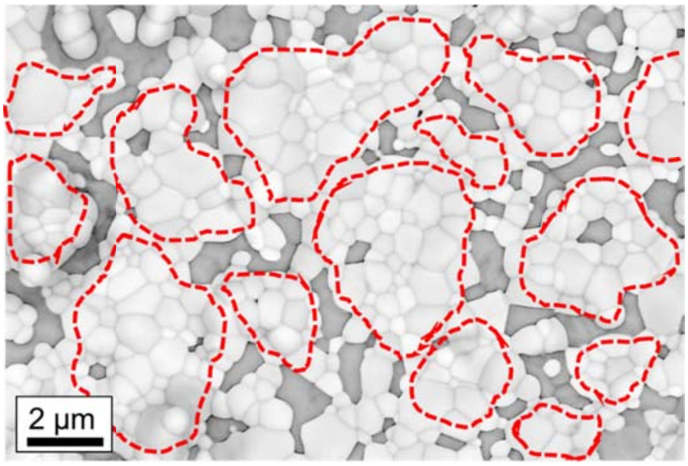

Supplementary Figure 4. SEM micrograph in back scattering mode of W-15 at% Cr with near-full density after sintering.

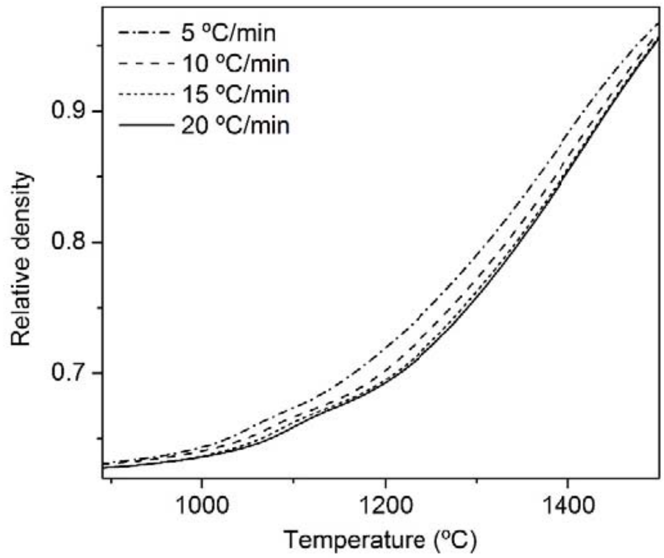

Supplementary Figure 5. Heating profiles of nanocrystalline W-15 at% Cr as function of temperature with 5, 10, 15, 20 °C/min heating rates.

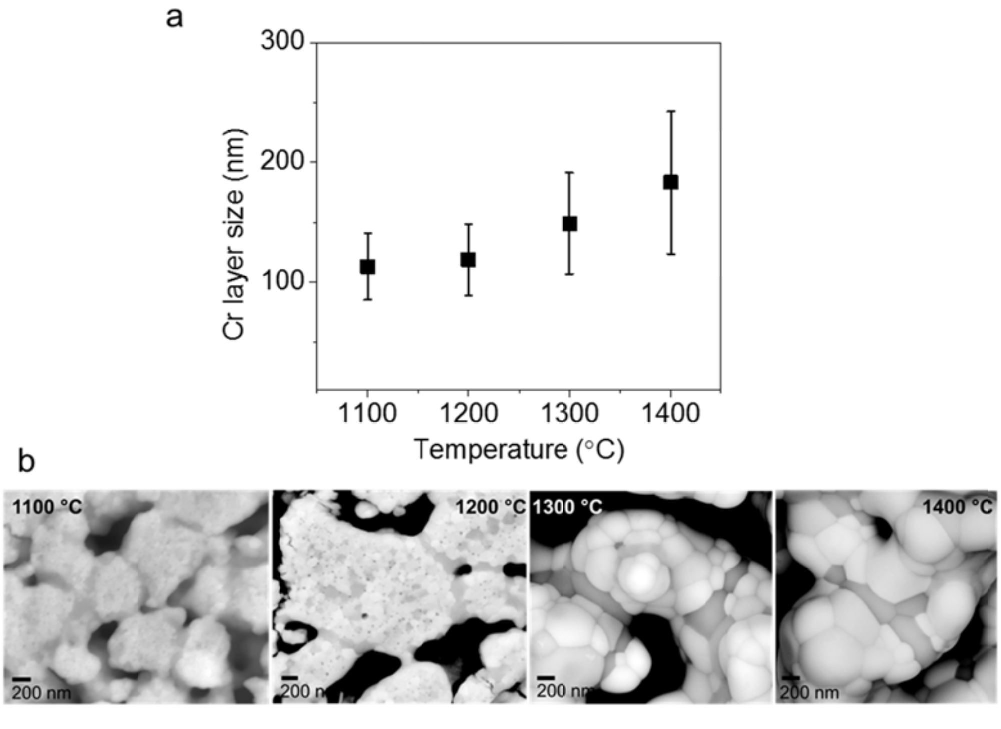

Supplementary Figure 6. a, The change in the size of the Cr-rich layer over sintering with increasing temperature. **b,** SEM micrographs in back-scatter mode of W-15 at% Cr with various temperatures.

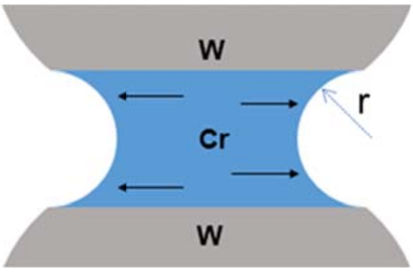

Supplementary Figure 7. A model geometry for an interparticle neck during nano-phase separation sintering. The blue region is the crystalline Cr phase and the W particles are grey.

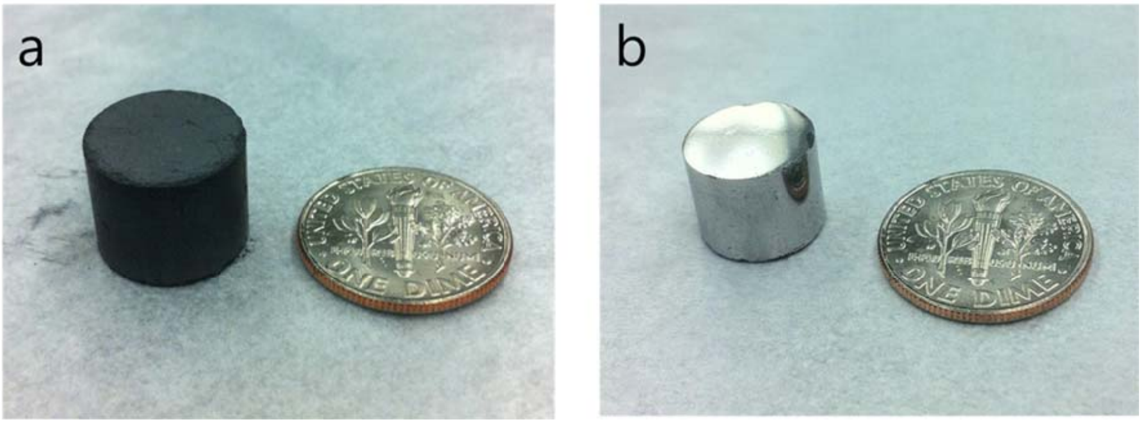

Supplementary Figure 8. A bulk sample of W-15 at% Cr **(a)** before sintering, in the as-pressed condition and **(b)** after pressureless sintering to >98% relative density and surface cleaning.

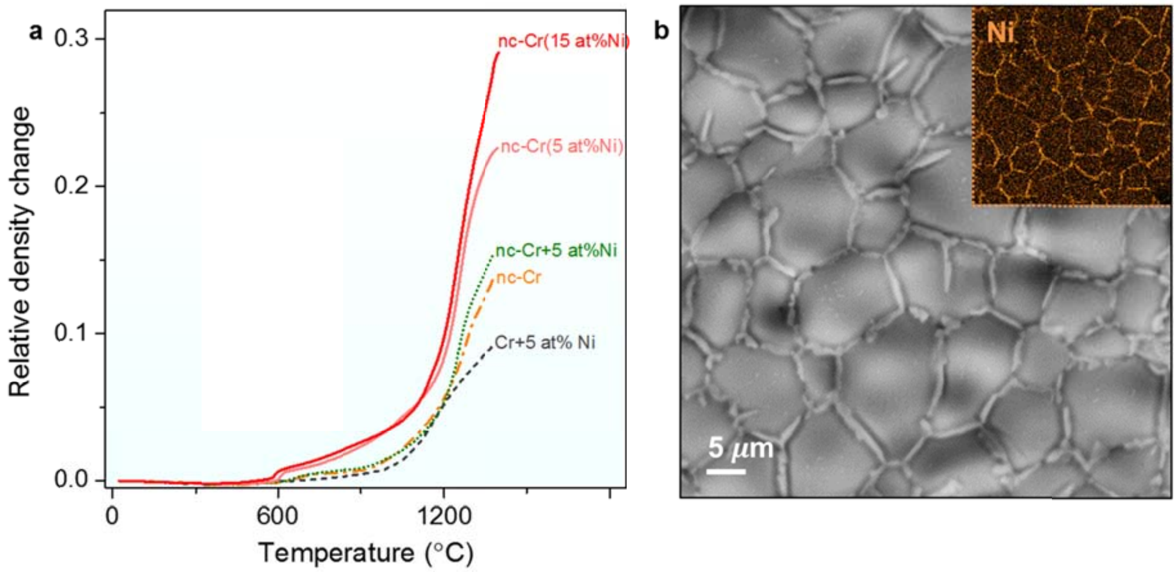

Supplementary Figure 9. a, Relative density changes of several Cr-Ni compacts as function of temperature indicate that supersaturation, phase separation and a fast transport layers are all required to achieve nano-phase separation sintering. **b,** Scanning electron microscopy (SEM) image of Cr (15 at% Ni) taken in back-scatter mode after sintering at 1200 °C delineates precipitated nickel around chromium necks, acting as a fast transport layers, with the inset of Ni elemental map using energy dispersive spectroscopy (EDS).

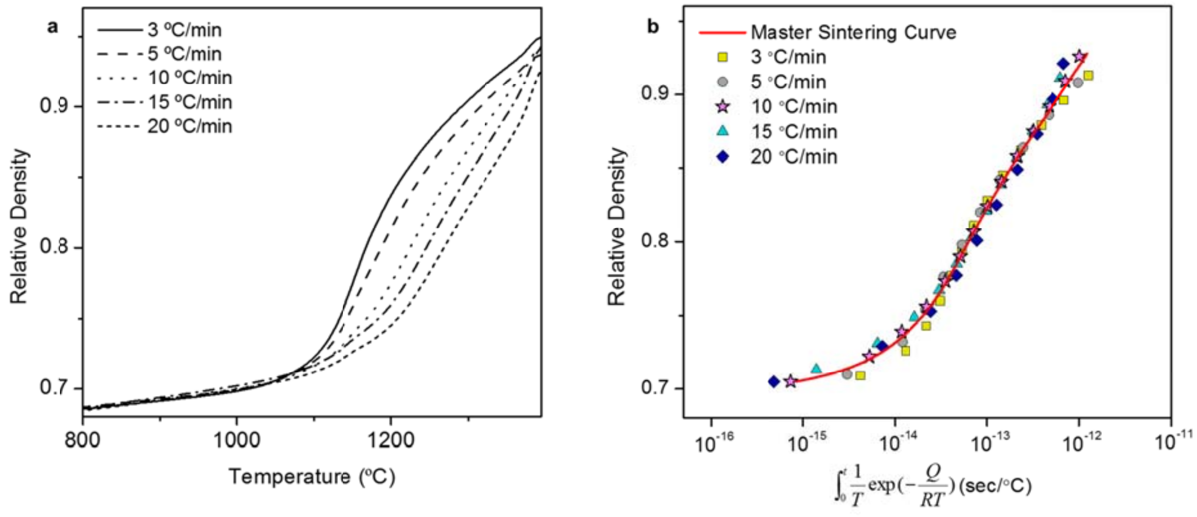

Supplementary Figure 10. a, Heating profiles of nanocrystalline Cr-15 at% Ni as function of temperature with 3, 5, 10, 15, and 20 °C/min heating rates. **b,** Master sintering curve and heating profiles of Cr-15 at% Ni, showing that all of the heating profiles collapse onto a single curve at a sintering activation energy of 258 kJ/mol.

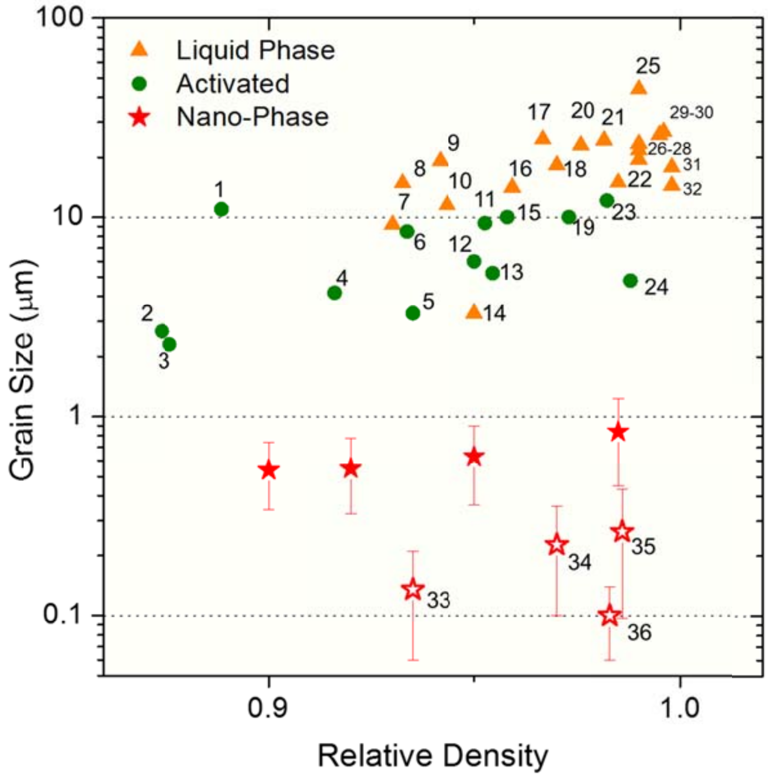

Supplementary Figure 11. Index numbers corresponding to Figure 4 of the paper.

Supplementary Table 1. Particle sizes of all control experiments including Cr-Ni system.

Sample	Mean Size (µm)	Mode Size (µm)	Standard Deviation (µm)
nc-W(Cr)	1.25	0.94	0.74
nc-W	2.30	1.85	0.96
nc-W+15 at% Cr	1.09	1.07	0.71
nc-W+15 at% nc-Cr	2.32	1.61	1.49
Pure Cr	6.38	6.26	2.78
W(Cr)	4.99	5.45	2.30
W+15 at% Cr	3.35	2.77	1.42
nc-Cr(15 at% Ni)	4.75	4.78	2.22
nc-Cr(5 at% Ni)	5.09	4.79	2.33
nc-Cr	5.21	4.17	2.61
nc-Cr+5 at% Ni	3.41	2.79	2.35
Cr+5 at% Ni	5.06	4.79	3.17

Supplementary Table 2. Data and References corresponding to Supplementary Figure 11.

Number	Materials (at.%)	Average initial particle size (μm)	Average sintered grain size (μm)	Density	Ref.
1	W-1Ni	4~6	11	0.889	8
2	W-17Fe	0.2	2.68	0.874	9
3	W-8.4Ni-3.6Fe	~6	2.3	0.876	10
4	W-6.3Fe	0.2	4.17	0.916	9
5	W-8.4Ni-3.6Fe	~6	3.3	0.935	10
6	W-6Fe-5.8Ni	0.2	8.48	0.934	9
7	W-19Cu-7.7Ni	~11	9.21	0.930	11
8	W-17.9Ni -9.5Cu	~9	14.87	0.933	11
9	W-17.9Ni -9.5Cu	~9	19.25	0.942	11
10	W-19Cu-7.7Ni	~11	11.59	0.943	11
11	W-3.2Fe-3Ni	0.2	9.35	0.953	9
12	W-0.9Co	0.7	6	0.95	12
13	W-3.2Fe	0.2	5.24	0.955	9
14	W-22Cu-2.6Ni	0.5	3.3	0.95	13
15	W-16.7Ni	0.2	10.03	0.958	9
16	W-19Cu-7.7Ni	~11	14.17	0.959	11
17	W-17.9Ni -9.5Cu	~9	24.7	0.967	11
18	W-19Cu-7.7Ni	~11	18.35	0.97	11
19	W-6Ni	0.2	10.03	0.973	9
20	W-17.9Ni -9.5Cu	~9	23.1	0.976	11
21	W-19Cu-7.7Ni	~11	24.47	0.982	11
22	W-3.2Fe-3Ni	1	15	0.985	14
23	W-3.1Ni	0.2	12.16	0.982	9
24	W-8.4Ni-3.6Fe	~6	4.8	0.988	10
25	W-3.2Fe -3Ni	5	44	0.99	14
26	W-11.9Ni-5.1Fe	~6	19.6	0.99	10
27	W-8.4Ni-3.6Fe	~6	21.8	0.99	10
28	W-4.9Ni-2.1Fe	~6	23.5	0.99	10
29	W-11.1Ni-5Fe	~3	26	0.995	15
30	W-18Ni-8Fe	-	27	0.996	16

31	W-6Mo-17.5Ni-7.9Fe	-	17.9	1.00	16
32	W-11.9Mo-17Ni-7.6Fe	-	14.5	1.00	16
33	W-30Ti-10Cr	-	0.135	0.935	
34	W-15Ti-15Cr	-	0.227	0.97	
35	W-20Ti-15Cr	-	0.264	0.986	
36	W-35Ti-10Cr	~4.5	0.1	0.983	

Supplementary Note 1

A dark-field STEM image of W-15 at% Cr heated to 1400 °C is shown in Supplementary Figure 3a, illustrating a structure of nanoscale W-rich grains with a Cr-rich grain delineated by a yellow dashed line. This image shows the occurrence of phase separation in the bulk, which complements the observations of phase separation on the powder particle surfaces and necks in Fig. 1 in the main article, and is expected based on the phase separation tendencies in the W-Cr system. We have presented similar nanostructural observations of the interior of powder particles in the same alloy system in Ref. 16 for additional comparison.

Supplementary Figure 3b presents the microstructure of W-15 at% Cr alloy heated to 1300 °C. This high resolution TEM image shows atomic-level interfacial structures between tungsten and the chromium-rich layer. Both phases are crystalline without any suggestion of a significant disordered region, such as might be expected in conventional activated sintering. We also examined the microstructure of W-15 at% Cr at nearly full density after sintering as shown in Supplementary Figure 4. We have circled in red those regions that are likely the original powder particles, and which are now W rich, having ejected Cr from solution. Such particles tend to be decorated around their perimeter with Cr-rich domains. The structure here is consistent with those we presented in the paper at lower densities and in Supplementary Figure 6b.

Supplementary Note 2

The integral of instantaneous linear shrinkage rate during sintering can be represented as follows[1]:

$$\int_{\rho_0}^{\rho} \frac{(G(\rho))^n}{3\rho\Gamma(\rho)} d\rho = \int_0^t \frac{\gamma\Omega D_0}{kT} \exp(-\frac{Q}{RT})\, dt \quad (1)$$

where γ is the surface energy, Ω is the atomic volume, R is the gas constant, T is the temperature, G is the average grain size, ρ is the relative density, t is time, Γ is a parameter which relates the driving force,

mean diffusion distance, and other geometric features of the microstructures, $D_0 = (D_v)_0$ and n = 3 for volume diffusion, and $D_0 = (\delta D_g)_0$ and n = 4 for grain-boundary diffusion. With slight rearrangement, Supplementary Equation 1 is divided into two parts:

$$\Phi(\rho) = \frac{k}{\gamma \Omega D_0} \int_{\rho_0}^{\rho} \frac{(G(\rho))^n}{3\rho \Gamma(\rho)} d\rho \quad (2)$$

which comprises all microstructural and materials properties except for activation energy.

$$\Theta(t, T(t)) = \int_0^t \exp(-\frac{Q}{RT})\, dt \quad (3)$$

which relies only on Q and the heating time-temperature profile. The activation energy can be estimated by Supplementary Equation 3; the correct activation energy, Q, will make all of the data computed through Supplementary Equation 3 collapse onto a single curve. For assessing the sintering activation energy of nanocrystalline W-15 at% Cr, heating profiles at 5, 10, 15, and 20 °C/min shown in Supplementary Figure 5 were employed. The results are shown in Fig. 3 in the manuscript.

Supplementary Note 3

In some enhanced densification methods such as liquid phase sintering, the formation of the second phase permits mechanical densification by flow, i.e., mechanical deformation, of the softer (liquid) phase. This leads to a significant microstructure evolution as a function of density. To verify that nanophase separation sintering does not exhibit such structural evolution, we measured the size of the Cr-rich necks between powder particles for several sintered densities. The results are shown in Supplementary Figure 6. The Cr-rich layer does not change beyond the measurement uncertainty over the range of relevance of the conditions. Moreover, the trend in Supplementary Figure 6a shows, if anything, an increase in the Cr

layer thickness; unlike liquid phase sintering where densification might lead to flow and compression of these layers, no such trend is observed here.

Supplementary Note 4

The driving force for sintering in the case of nano-phase separation sintering can be estimated using prior models, e.g., that for liquid phase sintering. A model geometry is shown in Supplementary Figure 7, and corresponds to the one shown in Supplementary Figure 6b or the schematic in Fig. 3 of the paper. (Note that there is no liquid phase in the present work, and instead the phase between two particles in the model is a crystalline Cr phase that is a rapid diffusion pathway for tungsten.)

The driving force for sintering can be represented by the chemical potential gradient, $\nabla(\mu_w - \mu_v)$ where μ_w, μ_v are the chemical potentials of a W atom and of a vacancy, respectively and the capillary pressure at the neck provides the gradient. Therefore the driving force for sintering is finally estimated as $-\frac{\alpha\Omega_w\gamma_{cr}}{r}$ where α is a constant, Ω_w is the atomic volume of W, γ_{cr} is the surface energy of Cr, and *r* is the radius of pore as delineated in Supplementary Figure 7.

If the kinetically limiting mass transport process leading to densification is assumed to be W diffusion through Cr, the densification rate calculated using the equation of the driving force above based on the microstructural geometry that DeHoff has provided[2] is as follows: $-\frac{dL}{Ldt} = \frac{\gamma\Omega_w}{kT}\left(\frac{\Gamma_b\delta D_b}{G^4}\right)$ where L is the length of the compact, γ is the surface energy, Ω_w is the atomic volume of W, k is the Boltzmann constant, *T* is the temperature, *G* is the average particle size, *t* is time, δ is the thickness of the Cr-rich layer, D_b are the coefficients for W diffusion in Cr, and Γ is a parameter which relates the driving force, mean diffusion distance, and other geometric features of the microstructure[3]. Introducing reasonable values for each parameter at the onset of sintering into the right-hand side of this equation (γ ~ 2.3 J/m^2 [4], $\Gamma_b \sim 10^4 - 10^5$, $D_b \sim 10^{-19}\ m^2/sec$ [5], G $\sim 10^{-6}\ m$, δ ~ 120 nm) results in ~$2.5 \times 10^{-7} - 2.5$

$\times 10^{-6}$/sec which has the same order of magnitude as the initial shrinkage rate $(-\frac{dL}{L\,dt})$ of nanocrystalline W-15 at% Cr that we experimentally measured, ~ 10^{-6}/sec, at the onset of sintering.

Supplementary References

1. Su H. H., Johnson D. L. Master sintering curve: A practical approach to sintering. *J Am Ceram Soc* **79,** 3211-3217 (1996).

2. DeHoff R. T. A Cell Model for Microstructural Evolution during Sintering. In: Kuczynski G, Miller A, Sargent G (eds). *Materials Science Research*. Springer US, 1984, pp 23-34.

3. Hansen J. D., Rusin R. P., Teng M. H., Johnson D. L. Combined-Stage Sintering Model. *J Am Ceram Soc* **75,** 1129-1135 (1992).

4. Murr L. E. *Interfacial phenomena in metals and alloys* (Addison-Wesley Pub. Co., Advanced Book Program, Reading, Mass., 1975).

5. Park M., Alexander K. C., Schuh C. A. Tungsten diffusion in chromium: Experiments and atomistic modeling. *J Alloy Compd* **611,** 433-439 (2014).

6. Monma K., Suto H., Oikawa H. Diffussion of Ni^{63} and Cr^{51} in nickelchromium alloys. *J. Jpn. Inst. Met* **28,** 188-192 (1964).

7. Mundy J. N. *et al.* Self-diffusion in chromium. *Phys Rev B* **24,** 658-665 (1981).

8. Gupta V. K., Yoon D. H., Meyer H. M., Luo J. Thin intergranular films and solid-state activated sintering in nickel-doped tungsten. *Acta Mater* **55,** 3131-3142 (2007).

9. Amato I. On the mechanism of activated sintering of tungsten powders. *Mater Sci Eng* **10,** 15-22 (1972).

10. Park S. J., Martin J. M., Guo J. F., Johnson J. L., German R. M. Grain growth behavior of tungsten heavy alloys based on the master sintering curve concept. *Metall Mater Trans A* **37A,** 3337-3346 (2006).

11. Kothari N. C. Densification and Grain Growth during Liquid-Phase Sintering of Tungsten-Nickel-Copper Alloys. *J Less-Common Met* **13,** 457-468 (1967).

12. Li C. J., German R. M. The Properties of Tungsten Processed by Chemically Activated Sintering. *Metall Trans A* **14,** 2031-2041 (1983).

13. Johnson J. L., German R. M. Solid-state contributions to densification during liquid-phase sintering. *Metall Mater Trans B* **27,** 901-909 (1996).

14. Park J. K., Kang S. J. L., Eun K. Y., Yoon D. N. Microstructural Change during Liquid-Phase Sintering of W-Ni-Fe Alloy. *Metall Trans A* **20,** 837-845 (1989).

15. Lee W. S., Chan T. Y. Microstructural Evolution and Mechanical Properties under High Strain Rate Testing of W-3.99Ni-1.71Fe Sintered by a Two-Stage Sintering Process. *Mater Trans* **53,** 1318-1323 (2012).

16. Bose A., German R. M. Microstructural Refinement of W-Ni-Fe Heavy Alloys by Alloying Additions. *Metall Trans A* **19,** 3100-3103 (1988).
